# Nanochannel-Confined TAMRA-Polypyrrole Stained DNA Stretching by Varying the Ionic Strength from Micromolar to Millimolar Concentrations

**DOI:** 10.3390/polym11010015

**Published:** 2018-12-22

**Authors:** Seonghyun Lee, Yelin Lee, Yongkyun Kim, Cong Wang, Jungyul Park, Gun Young Jung, Yenglong Chen, Rakwoo Chang, Shuji Ikeda, Hiroshi Sugiyama, Kyubong Jo

**Affiliations:** 1 Department of Chemistry and Integrated Biotechnology, Sogang University, Seoul 04107, Korea; shlee@sogang.ac.kr (S.L.); godl20141300@gmail.com (Y.L.); ykkim1228@naver.com (Y.K.); 2 Department of Mechanical Engineering, Sogang University, Seoul 04107, Korea; wang.tsung@gmail.com (C.W.); sortpark@sogang.ac.kr (J.P.); 3 School of Material Science and Engineering, GIST, Gwangju 61005, Korea; gyjung@gist.ac.kr; 4 Institute of Physics, Academia Sinica and Department of Chemical Engineering, National Tsing-Hua University and Department of Physics, National Taiwan University, Taipei 10617, Taiwan; ylchen@gate.sinica.edu.tw; 5 Department of Chemistry, Kwangwoon University, Seoul 01897, Korea; rchang@kw.ac.kr; 6 Department of Chemistry, Graduate School of Science, Kyoto University, Sakyo-Ku, Kyoto 606-8501, Japan; ikeda.shuji.2a@kyoto-u.ac.jp (S.I.); hs@kuchem.kyoto-u.ac.jp (H.S.)

**Keywords:** DNA stretching length, persistence length, nanochannel

## Abstract

Large DNA molecules have been utilized as a model system to investigate polymer physics. However, DNA visualization via intercalating dyes has generated equivocal results due to dye-induced structural deformation, particularly unwanted unwinding of the double helix. Thus, the contour length increases and the persistence length changes so unpredictably that there has been a controversy. In this paper, we used TAMRA-polypyrrole to stain single DNA molecules. Since this staining did not change the contour length of B-form DNA, we utilized TAMRA-polypyrrole stained DNA as a tool to measure the persistence length by changing the ionic strength. Then, we investigated DNA stretching in nanochannels by varying the ionic strength from 0.06 mM to 47 mM to evaluate several polymer physics theories proposed by Odijk, de Gennes and recent papers to deal with these regimes.

## 1. Introduction

The DNA molecules are polyelectrolyte chains that exhibit unique electrostatic properties that make their conformations dependent upon the ionic strength [1,2]. Precise biophysical properties are essential parameters to understand DNA chains’ behavior in a nano/microfluidic device [3]. Pioneers in the field of polymer physics predicted geometrically confined polymer conformation and its behavior much earlier than experimental observation was possible [4,5]. Microscopic visualization of an individual DNA molecule in a nano-/microfluidic device has provided a powerful tool for the study of polymer physics to evaluate theoretical predictions developed over several decades [6,7,8,9,10,11,12,13,14]. 

This visualization essentially requires DNA staining reagents. Among many DNA staining dyes, YOYO-1, an oxazole yellow homodimer [15], has been widely used for this purpose [16]. This dye intercalates into the stacked base pairs with a high-intensity contrast [17], showing nearly homogeneous fluorescence along the DNA backbone [18]. However, YOYO-1 stained DNA has several undesirable features. First, YOYO-1 unwinds double-stranded DNA to make partially melted single-strand that has entirely different physical properties from the original double-stranded DNA, particularly for the persistence length and the contour length [19]. Besides, single-strands tend to break more frequently than double-strands. This issue becomes more serious when exposed to bright laser light. Thus, light-induced DNA cleavage has been a notorious problem in using the intercalating dyes [20]. The chance of DNA cleavage increases for tethered or nanochannel-confined DNA, because there exist additional mechanical and thermodynamically pulling forces in such environments [21].

YOYO-1 is known to increase the contour length of DNA but there has been a controversy over how the contour length varies with the dye [22,23]. Kundukad et al. systematically compared nine papers published from 1997 to 2014, which had reported different contour lengths from 118% to 150% when compared with native B-form DNA [24]. The longest contour length reported so far was 24.67 µm for YOYO-1 stained λ DNA, while the B-form λ DNA should be 16.3 µm long (48,502 × 0.337 nm) [19]. As for the persistence length, many authors have assumed a linear increase from 50 nm up to 65 nm with increasing staining ratios of YOYO-1 molecules per DNA base pairs [25]. However, Künther et al. reported that the persistence length does not increase with YOYO-1 staining ratios, although it was not the same as that of the natural B-form DNA [26]. Different persistence lengths have been reported, with the range from 11.8 nm to 66 nm for YOYO-1 stained DNA [24].

Recently, we have developed several novel DNA staining reagents, such as fluorescent protein-DNA binding peptides (FP-DBPs) [21,27,28] and TAMRA-polypyrrole [29]. They consist of a fluorophore and a DNA binding moiety, which makes the fluorophore well separated from the DNA backbone. Thus, none of them cause light-induced photocleavage. More importantly, they do not increase the DNA contour length because most of them bind the DNA groove without intercalation. Accordingly, we believe that DNA molecules stained with these novel staining reagents would provide a useful tool to evaluate the predictions of polymer physics theory explicitly. Among them, TAMRA-polypyrrole would be ideal because it is a minor groove binder and it works well over a broad range of ionic strengths with consistent staining property, whereas FP-DBPs are dissociated from DNA at elevated ionic strength. In addition, TAMRA-polypyrrole has a neutral charge and is far smaller than FP-DBP, which may be critical for nanochannel confinement.

Here we experimentally measured the persistence length using surface-tethered DNA within a flow cell at various ionic strengths under quiescent conditions. Our measured persistence length (*p*) showed the dependence of the inverse square-root of ionic strength (√*I* ) that agrees well with the Dobrynin theory [30]. Then, based on our measured persistence lengths, we investigated various nanochannel confined DNA conformation regimes from Odijk’s regime [31] to de Gennes regime [32]. Our measurements showed three regimes with two stepwise transitions. We interpreted these data with well-known theories such as classical Odijk, backfolded Odijk, de Gennes and extended de Gennes theories.

## 2. Materials and Methods

### 2.1. Materials

T4GT7 DNA was purchased from Nippon gene (Tokyo, Japan) and *λ* DNA was from New England Biolabs (Ipswich, MA, USA). Polydimethylsiloxane (PDMS) and its curing agent (Sylgard 184) were from Dow Corning (Midland, MI, USA). Other chemicals were purchased from Sigma-Aldrich (St. Louis, MO, USA).

### 2.2. Synthesis of TAMRA-Polypyrrole

TAMRA-polypyrrole was synthesized in the lab as previously described [28,32]. Briefly, a computer-assisted Fmoc solid–phase synthesis of polyamides was performed from Fmoc-Py-oxime resin and cleavage from the resin was performed with 1 mL of 3-(dimethylamino) propylamine (Dp) or 3,3’-diamino-N-methyldipropylamine treatment for 3 h at 55 °C. The crude products were purified by flash chromatography and the collected fractions were lyophilized to collect the objective compounds. The purified polypyrrole (1.3 mg, 1.0 × 10^−3^ mmol) and 5–TAMRA NHS ester (1.2 mg, 2.3 × 10^−3^ mmol) were dissolved in DMF (190 µL) and DIEA (0.70 µL, 4.0 × 10^−3^ mmol), followed by mixing at room temperature with shielding the light. After checking this reaction had been finished, the reaction mixture was purified by reversed–phase HPLC, followed by lyophilization of the collected fractions to afford TAMRA-polypyrrole (1.7 mg, 1.0 × 10^−3^ mmol, quant) as a purple powder.

### 2.3. Flow Chamber

A flow chamber was prepared as described and illustrated with a video in a previous paper [33]. Briefly, an acid-cleaned coverslip was placed on custom-made acrylic support with a height of 100 μm by double-sided tape. Then 40 µg/mL of biotinylated BSA, 25 µg/mL of Neutravidin and 1 µM of *λ* DNA overhang oligo (5’-*p*-GGGCGGCGACCT-Triethyleneglycol-biotin-3’) were sequentially loaded into the flow chamber and each was incubated for 10 min at room temperature. After the surface preparation, *λ* DNA and T4 DNA ligase were added and incubated at room temperature for 30 min. After washing the remaining enzyme mixture with diluted buffer, the diluted TAMRA-polypyrrole solution flowed into the channels, resulting in visualization of the tethered DNA. Stained DNA molecules were visualized under a continuous flow of diluted buffer with the flow rate at 5 μL/min.

### 2.4. Preparation of T4 DNA for Nanochannel

T4GT7 DNA stock solution (~0.38 µg/µL) was diluted to a final concentration of 3.5 ng/μL in diluted buffer (adding deionized water to 1 × TE, 10 mM Trizma base and 1 mM EDTA, pH 8.0) and TAMRA-polypyrrole was diluted to a final concentration of 0.7 µM in the same buffer solution. Then DNA and dye solutions were mixed 1:1 as volume ratio, followed by incubation for 30 min at room temperature with light protection. Buffers and DNA samples were prepared immediately before experimental use. Each ionic strength of the buffer solution was determined by the comparison to the conductivity (CON510, EUTECH, Seoul, Korea) of sodium chloride solutions like our previous publications [9,11]

### 2.5. DNA Loading into PDMS Nanochannels

PDMS nanochannels were fabricated from a replica of a silicon wafer mold that was previously made (250 nm × 250 nm) [9]. The PDMS base solution mixed with the curing agent (10:1 w/w ratio) was poured on the patterned wafer and cured at 65 °C for 4 h or longer. After curing the PDMS device, it was treated for 30 s in an air plasma generator (Cute Basic, FemtoScience, Suwon, Korea) to make the surface hydrophilic and stored in deionized water before use. A PDMS device was mounted on a piranha-cleaned cover glass (22 mm × 22 mm) [34] and 2 μL of the DNA-dye mixture sample was loaded into the PDMS nanochannel device and incubated for 5 min. The buffer solution with the same ionic strength was then added around the PDMS nanochannel in a plastic well. Copper electrodes were dipped into a buffer and an electrical field (15–50 V across 25 mm) was applied to load DNA molecules into the nanochannels.

### 2.6. Microscopy

The microscopy system consisted of an inverted microscope (Zeiss Observer A1, AG; Zeiss, Oberkochen, Germany) equipped with a 63× Zeiss Plan-Neofluar oil immersion objective and was illuminated by an LED light source (SOLA SM II light engine, Lumencor, Beaverton, OR, USA). The light was passed corresponding filter sets (Semrock, Rochester, NY, USA), installed to prevent excitation light from reaching the camera. Fluorescence images were captured using a scientific complementary metal–oxide–semiconductor (sCMOS) camera (PRIME; Photometrics, Tucson, AZ, USA) with 100 ms exposure time and images were stored as 16-bit TIFF file using Micro-manager. ImageJ was utilized with the Java plug-in developed in our lab to measure DNA stretching [11].

### 2.7. Calculation of the Effective Diameter (w) of DNA

The effective diameter (*w*) listed in Table 1 was calculated using Equation (1) [35]:

(1) $$w=\kappa^{-1}\left[0.7704+\ln F\right]=\kappa^{-1}\left[0.7704+\ln\left(\frac{\pi }{2l_{B}\kappa }{\left(\frac{y_{0}}{\gamma K_{0}\left(x_{0}\right)}\right)}^{2}\right)\right]$$

where $x_{0}=\kappa a$ (*a* = 1.2 nm, representing the DNA radius), $y_{0}$ was the dimensionless surface potential, $K_{0}$ was the zeroth order modified Bessel functions of the second kind and γ was the correction factor defined as $\gamma =y_{0}/y_{DH}\left(x_{0}\right)$, where $y_{0}$ was the normalized surface potential and $y_{DH}\left(x_{0}\right)$ was the surface potential obtained from the Debye-Hückel equation. The normalized surface potential ($y_{0}$) could be determined by solving the Poisson-Boltzmann Equation [36]:

(2) $$\frac{1}{x}\frac{d}{dx}\left(x\frac{dy}{dx}\;\right)=\sinh y$$

where x=κr, with *r* representing the distance from the center and $y=e\psi /k_{B}T$, with ψ representing the electric potential in the ionic atmosphere. Assuming the DNA molecule as a cylinder with radius a (1.2 nm),$\;y_{0}=y\left(x_{0}\right)=e\psi_{0}/k_{B}\;T$, where $x_{0}=\kappa a=\sqrt{I}/0.304\times 1.2$. The boundary condition for solving the Poisson-Boltzmann equation was obtained from the Gauss’s electric flux theorem that provides a relationship between the DNA charge density (Ze=2×0.73e/0.337 nm) and the potential surface gradient [37]:

(3) $${\left(\frac{dy}{dx}\right)}_{x_{0}}=-\frac{2Ze^{2}}{x_{0}Dk_{B}T}=-\frac{2Zl_{B}}{x_{0}}=-\frac{1.565}{\sqrt{I}\;}$$

The numerical integration of the exact Poisson-Boltzmann equation followed the method described by Stigter, where the second order partial differential equation was converted into a set of coupled first order differential equations and then solved using the Runge-Kutta integration method. In practice, we initially chose a long distance $x_{i}$ and set $y\left(x_{i}\right)=10^{-3}$ and then we calculated ${\left(dy/dx\right)}_{x_{0}}$ by integrating Equation (2) from $x_{i}$ to $x_{0}$. We then iterated the value of $x_{i}$ using Equation (2) until Equation (3) is satisfied with a maximum error <$10^{-5}$. The determination of $x_{i}$ provided the numerical value of $y_{0}$ from the Poisson-Boltzmann solution.

For the correction factor $\gamma =y_{0}/y_{DH}\;\left(x_{0}\right)$, $y_{DH}\left(x\right)$ was obtained from solving the linearized Poisson-Boltzmann equation or the Debye-Hückel equation, which was given by $\frac{1}{x}\frac{d}{dx}\left(x\frac{dy_{DH}}{dx}\right)=y_{DH}$ with the boundary condition that $y_{DH}\left(x_{i}\right)=y\left(x_{i}\right)$ [36]. The Debye-Hückel potential, $y_{DH}\left(x\right)$, had the analytic functional form of $y_{DH}\left(x\right)=CK_{0}\left(x\right)$. $C=y\left(x_{i}\right)/K_{0}\left(x_{i}\right)$ was derived from the boundary condition of $y\left(x_{i}\right)=y_{DH}\left(x_{i}\right)=CK_{0}\left(x_{i}\right)$. Additionally, $y_{DH}\left(x_{0}\right)=CK_{0}\left(x_{0}\right)=\left(y\left(x_{i}\right)/K_{0}\left(x_{i}\right)\right)K_{0}\left(x_{0}\right)$. Since we had already determined $x_{0},y_{0}\;and\;x_{i}$ from setting $y\left(x_{i}\right)=10^{-3}$, *γ* could be determined as

(4) $$\gamma =\frac{y\left(x_{0}\right)}{y\left(x_{i}\right)}\frac{\;K_{0}\left(x_{i}\right)}{K_{0}\left(x_{0}\right)}$$

## 3. Results and Discussion

### 3.1. DNA Contour Length (L) 

**Figure 1:**
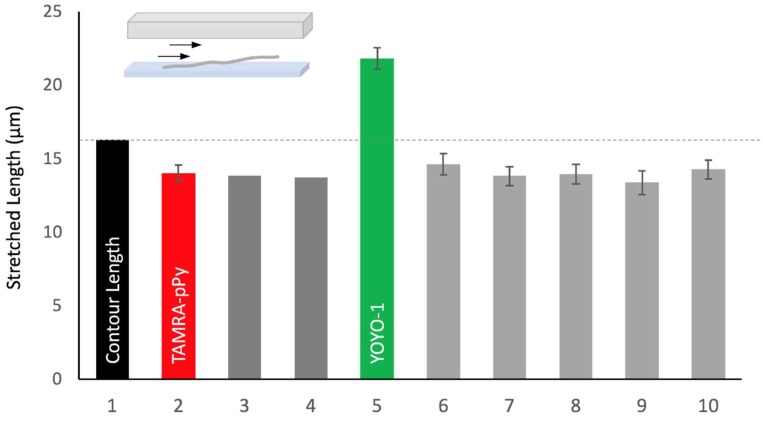
Comparison of surface-tethered DNA stretches. **1**: contour length, **2**: TAMRA-polypyrrole stained DNA, **3**: DNA stretch calculated from a graph from the paper by Bustamante et al., in which they stretched a natural B-form DNA by optical tweezers without DNA staining reagent [38]. **4**: DNA stretch predicted from our previous calculation of *F* = 0.79 pN [27]. **5**: YOYO-1 stained DNA, **6~10**: FP-DBP stained DNA (6: 2(KW)2, 7: KW5, 8: K6, 9: 2HMG, 10: 2(SPRK)) [27,33]. Flow rate = 100 µL/min.

Figure 1 compares surface-tethered DNA molecules dependent on the staining reagents. First of all, TAMRA-polypyrrole stained DNA (**2** in Figure 1) stretched up to 14.03 ± 0.54 µm (*X*/*L* = 86% ± 3%) at 100 µL/min within 100 µm high flow cell [29]. Previously, we calculated the force of 0.79 pN applied to the λ DNA in the same flow cell (100 µL/min) [27]. Based on this value, we checked Bustamante’s paper, in which they measured DNA stretches versus the force by optical tweezers. From their graph, we obtained 85% stretch (**3** in Figure 1), which agreed with our previous prediction of 84% (**4** in Figure 1). In contrast, YOYO-1 stained DNA (**5** in Figure 1) stretched up to 21.8 ± 0.72 µm (*X*/*L* = 134% ± 4.4%), which is significantly larger than other stretches. However, it is notable that this stretch would be 88% ± 3% compared with Murade’s contour length (24.67 µm) for YOYO-1 stained DNA [28]. For comparison, we added DNA stretches stained by FP-DBPs (DNA binding peptides) from **6** to **10** in Figure 1 [21,27]. Figure 1 suggests that TAMRA-polypyrrole stained DNA preserves the contour length of natural B-form DNA, while YOYO-1 stained DNA changes it considerably. Therefore, we would like to use the contour length (*L*) of natural B-form DNA for the remaining part of this paper, such as 16.3 µm for *λ* DNA (48,502 bp × 0.337 nm/bp) and 55.8 µm for T4 DNA (165,644 bp × 0.337 nm/bp). 

### 3.2. DNA Persistence Length (p)

**Figure 2:**
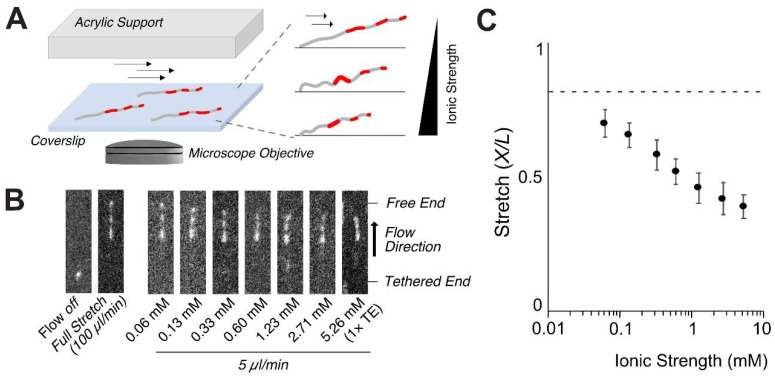
Ionic strength dependent DNA stretching: (**A**) Schematic illustration of surface-tethered DNA stretches and microscopic DNA images in a flow chamber; (**B**) Microscopic images of stretched DNA molecules dependent on the ionic strength at a reduced flow rate of 5 µL/min. The position of the tethered end was determined by flow-off images.; (**C**) DNA stretch ratio (*X*/*L*) vs. ionic strength. Each point represents more than 100 molecules with the standard deviation as an error bar.

Figure 2 demonstrates that DNA stretch varies according to the ionic strength. The persistence length (*p*) is dependent on the ionic strength (*I*). A well-known theory is the Odijk, Skolnick and Fixman equation (OSF equation) given by [39,40].

(5) $$p_{OSF}=p_{0}+0.0324/I\;\left(nm\right)$$

where *p*_0_ is the intrinsic persistence length (50 nm) [2]. However, the validity of this theory at low ionic strength has been debated. Dobrynin pointed out the limitations of the OSF equation [41] and suggested that OSF theory exaggerated the dependence of the electrostatic persistence length on the Debye length; thus, *p* should be proportional to *I*^−1/2^ instead of *I*^−1^. According to his opinion, strong electrostatic interactions between chain segments create a correlation hole with a size in the order of the Debye screening length (*κ*^−1^) around the chain backbone, which can weaken the effects of ionic strength on the persistence length [42]. Thus, Dobrynin proposed an empirical formula [30] from other previous experimental results [2,43].

(6) $$p_{Dob}=46.1+1.92/\surd I\;\;\left(\mathrm{nm}\right)\;$$

Although there have been several attempts to validate the accuracy of each theory associated with Equation (5) and Equation (6), the results have remained inconclusive because of the difficulty of measurements at very low ionic strengths [42,44,45].

The persistence length (*p*) can be experimentally measured using the following Equation [46].

(7) $$\frac{Fp}{k_{B}T}=\frac{1}{4}{\left(1-\frac{X}{L}\right)}^{-2}-\frac{1}{4}+\frac{X}{L}$$

where *F* is the pulling force, *X* is the apparent DNA stretch and *L* is the contour length. As we mentioned *X*/*L* = 0.86 and *F* = 0.79 pN at 100 µL/min in the 1 × TE buffer, we were able to determine p=69.7 nm for *I* = 5.26 mM (1 × TE) from Equation (7). This value is larger than 56.2 nm calculated from OSF (Equation (5)) but a little bit smaller than 72.5 nm from Dobrynin (Equation (6)). We attempted to validate which theory is more appropriate by measuring the persistence length over a wide range of the ionic strength. Figure 2B shows microscopic images of various DNA stretch ratios (*X*/*L*) at 5 µL/min ranged from 39% (1 × TE) to 70% (*I* = 0.06 mM) by varying the ionic strength. Since p=69.7 nm at 1 × TE, we calculated the force of 48 fN using Equation (7) from X/L=0.39 at 5 µL/min. We diluted 1 × TE buffer by 2, 5, 10, 20, 50, 100 times. We determined the ionic strength by measuring conductivity compared with that of NaCl solution as described in the method. For these diluted solutions, we calculated persistence lengths using Equation (7) from measured *X*/*L* (Figure 2b). These persistence lengths at different ionic strength allowed us to evaluate the validity of OSF theory versus Dobrynin theory.

**Figure 3:**
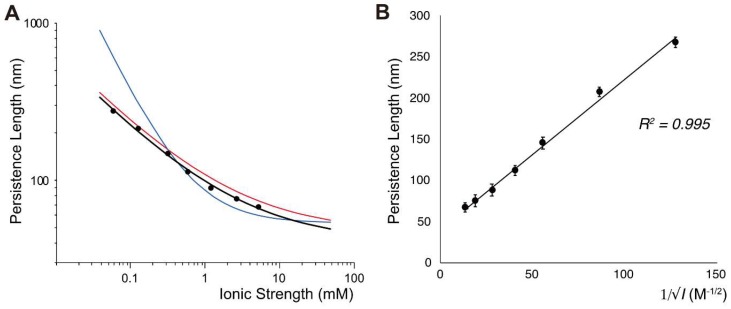
(**A**) Persistence lengths measured at various ionic strengths (Equation (7)) compared with Equation (5) (blue), Equation (6) (red) and the best fit (black).; (**B**) The linear correlation between *p* and $I^{-1/2}$ (Equation (8)) with *r*^2^ = 0.995.

Figure 3A shows the comparison of experimental data with Equation (5) (blue) and Equation (6) (red), implying that Dobrynin’s argument is more appropriate than the OSF theory. Figure 3B shows excellent linearity of the persistence length (p) with the dependence of *I*^−1/2^. Moreover, we deduced an empirical equation from our measurements as

(8) p=42.1+1.90/√I  (nm)

where *I* has the unit of molar concentration (M). According to Dobrynin’s paper, the persistence length should be inversely proportional to $\sqrt{I}$ [30]. He obtained numerical coefficients from two other experimental papers [2,43]. It is noticeable that there is a slight difference from Equation (7) and Equation (8). This difference may be attributed to Dobrynin’s original derivation for Equation (6), in which he only used a dataset for extensible worm-like chain model from Baumann’s data [2], which correspond to the pulling force stretched DNA longer than B-form contour length (X>L). For two other different DNA models from Baumann et al. [2], we would get p=45.0+1.48/√I with $r^{2}=0.752$ for the strong-stretch limit (X→L) and p=39.9+1.97/√I with $r^{2}=0.753$ for the inextensible wormlike chain (X<L), which is more relevant to our dataset in Figure 2 and Figure 3 (*X* = 6.4~11.5 µm). If we combine all data points in Figure 3 of Ref. [30] that included two experimental datasets [2,43], the equation would be p=43.0+1.87/√I with $r^{2}=0.780$. We believe that Equation (8) is more appropriate to analyze our data because our data covered a wider range of the ionic strength from 0.06 mM and showed better correlation ($r^{2}=0.995$, Figure 3B) than Dobrynin’s consideration ($r^{2}=0.887$) [30]. Therefore, we used Equation (8) to calculate the persistence length for the following analysis in this paper.

### 3.3. TAMRA-Polypyrrole Stained DNA in Nanochannel

**Figure 4:**
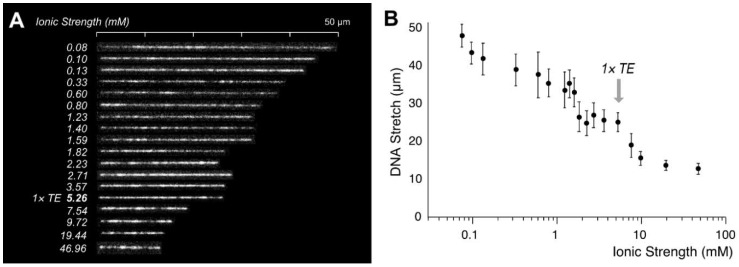
Nanochannel confined DNA stretches depending on the ionic strength: (**A**) T4GT7 DNA (165,644 bps, *L* = 55.8 µm) confined in a 250 × 250 nm nanochannel from 0.08 to 47 mM.; (**B**) The graph illustrates apparent DNA lengths. Each point represents measurement from 50 to 250 DNA molecules with the standard deviation as an error bar. 16bit clip images in supporting information.

Now, we have both correct persistence length (*p*) and contour length (*L*). Based on these values, we attempted to re-investigate DNA stretching in a nanochannel to evaluate polymer physics theory [9]. Figure 4 demonstrates that DNA stretches decrease with the increase of the ionic strength from 0.075 mM (47.2 μm, 85%, *p* = 262 nm, *w* = 224 nm) to 47 mM (12.5 μm, 22%, *p* = 51 nm, *w* = 8 nm). In general, the DNA stretch decreases by the increase of ionic strength but it also has more complications. There exist three regimes with two abrupt transitions in the slope (*I* = 1.5 mM, *p* = 90 nm and *I* = 7.5 mM, *p* = 64 nm).

For this experiment, we used T4 DNA instead of *λ* DNA, because high A/T content in T4 DNA provided more precise visualization of entire molecules. TAMRA-polypyrrole generally stains AT-rich regions specifically via hydrogen bonding on minor groove [29]. Therefore, *λ* DNA is not an appropriate model molecule because the first half of the *λ* DNA has CG-rich regions, showing dim DNA backbone (see Figure 2B) [29], which is practically unfavorable in a nanochannel. However, DNA stretch is independent on the length and species. More importantly, we observed that DNA stretching ratios (*X*/*L*) were the same for λ DNA and T4 DNA at the same condition in a nanochannel as they were in our previous study of YOYO-1 DNA [8].

**Figure 5:**
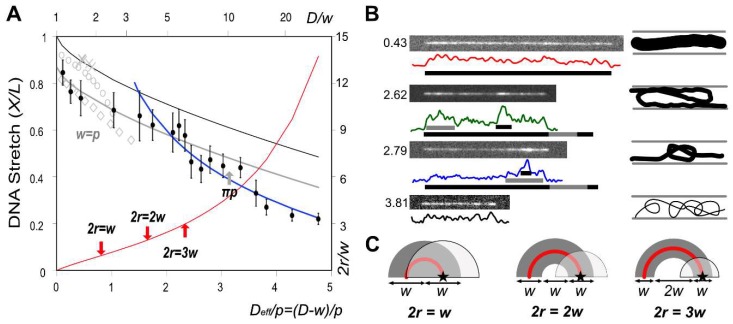
DNA stretch ratio (*X*/*L*) dependent on *p* and *w*: (**A**) The black line was drawn by Equation (11) and the gray line was down-shifted by 0.13 (Equation (12)). The gray symbols were obtained from other references: *X*: 38 nm nanochannel experiment [49]; open circles: simulation for p=4w; open diamond: simulation for p=w [50]. The blue line was the best fit for the de Gennes regime (Equation (14)). The red line represents *2r/w* where *r* is the hairpin radius (Equation (13)); (**B**) Microscopic DNA images and its intensity profiles at four data points representing the unfolded, backfolded, knotted and random chains. The corresponding *D*_eff_/*p* is also shown on the left side of each image; 16bit clip images in supporting information. (**C**) Illustrations depict three cases for the ratio of the hairpin diameter, *2r*, to the effective diameter, *w*. Stars and partially transparent semi-circles represent the DNA position and electrostatically repulsive regions defined by the effective diameter (*w*), respectively.

Figure 5 illustrates how the DNA stretch ratio (X/L) depends on both *p* and *w* in order to interpret them with polymer physics theories. Nanochannel-confined DNA molecule has been studied in many theories, simulations and experiments, which have been reviewed multiple times [10,12,47]. For fully elongated DNA molecules, Odijk introduced a theory to describe DNA conformation in a cylinder with diameter D≪p [5]. Under this strong confinement regime, a worm-like DNA chain would be deflected back and forth by the cylinder boundary. He defined the deflection segment length, λ=cos(θ) which had a scaling relation of $\langle \theta^{2}\rangle ≅{\left(D/P\right)}^{2/3}$. Later, he derived an analytical equation to explain our previous experimental observations of DNA stretching dependent on varying ionic strengths in a rectangular shape of nanochannel [8].

(9) $$\begin{array}{c} \frac{X}{L}=\cos\left(\theta \right)=1-\frac{1}{2}\langle \theta^{2}\rangle +\ldots =1-\frac{1}{2}\langle \theta_{x}^{2}\rangle -\frac{1}{2}\langle \theta_{y}^{2}\rangle +\ldots \\ ≅1-0.085\left({\left(\frac{A}{p}\right)}^{\frac{2}{3}}+{\left(\frac{B}{p}\right)}^{\frac{2}{3}}\right) \end{array}$$

where A and B are width and height of nanochannels. For square nanochannels, DNA stretch (*X*) could be simplified as

(10) $$X/L=1-2\alpha {\left(D/p\right)}^{2/3}$$

where α was recalculated as 0.09137 by hard-wall simulation, instead of 0.085 by Odijk’s harmonic potential approximation [48]. The first term of ‘1’ was originated from Taylor series to approximate cos(*θ*) when *θ* is very small. Physically, ‘1’ represents the rod limit without the effect of thermal fluctuation. This equation predicts that X=L when D=0, which forecast a problematic case that DNA cannot reach the full stretching even in 2 nm nanochannel. Furthermore, DNA has the effective diameter (*w*) due to the electrostatic repulsive force. It is crucial when *w* is considerably large enough to reduce the freedom of the worm-like DNA chain in the nanochannel. Therefore, the effective nanochannel dimensions ($D_{eff}$) should be defined to be D−w.

(11) $$X/L=1-2\alpha {\left(\left(D-w\right)/p\right)}^{2/3}$$

From this equation, the upper bound of w (or the lower bound of I) can be determined as w<D, because it is not plausible to load DNA into a nanochannel when w>D. We experimentally observed this upper bound. For instance, the lowest ionic strength was 0.061 mM in Figure 2 and Figure 3 and we could not load such DNA into a 250 nm square nanochannel at 0.061 mM because of D=w=250 nm. The lowest ionic strength for successful loading was 0.075 mM (w=225 nm<D), although we only observed a small number of DNA molecules that managed to enter the nanochannels at each loading.

The next question is that the term ‘1’ is valid in w=D, particularly for large *w*. Unfortunately, the prediction of Equation (11) (black line) was systematically higher than our measurements as shown in Figure 5A. As mentioned earlier, 2 nm nanochannel would stretch a DNA molecule up to its full contour length but this assumption may not be valid because *w* is the effective diameter including electrostatic interactions and the electrostatic repulsive force may not be strong enough to prevent the deflection of the DNA chain completely. Effective diameter has a shape of Boltzmann distribution rather than a solid cylindric rod. Our observation seems related to Chen’s recent simulation result [50]. In his figure, DNA stretch cannot reach the full stretching (X/L<1) when D−w→0. As shown as open gray diamonds in Figure 5A, his simulation predicted that the stretch might approach 85% when D→w=p and his prediction reached 94% when D→w=p/4 (open gray circles). By reducing w/p ratios to 1/4^9^, his simulation predicted X/L approaches the unity more closely. On the other hand, Bhandari et al. recently reported X/L = 90.39% in 38 nm square channels when *IS* = 16.9 ~ 72.2 mM, *w/p* = 0.13 ~0.22 [49]. Their results follow Equation (11) as shown as shown as *X* symbols in Figure 5A. 

The maximum stretch in our measurement was 0.85 when w/p=0.85 and $D_{eff}/p=0.10$. To fit our experimental data, we calculated the intercept by linear regression, which gave 0.87; thus, the relationship would be

(12) $$X/L=0.87-2\alpha {\left(\left(D-w\right)/p\right)}^{2/3}$$

The experimentally obtained gray line (Equation (12)) covers nine data points up to $D_{eff}/p\leq 2.33$ and the predicted values are a little bit higher for five more points ($2.4<D_{eff}/p<3.4$). The remaining four more points deviate significantly from the gray line ($3.4<D_{eff}/p$). There has been a dispute about the lower boundary condition for the Odijk’ regime. According to the original assumption and some previous studies, the Odijk regime is only valid for D≤p (Figure 5) [47,51]. Other papers claimed that it should be the Kuhn length (D≤2p) [52,53]. Other boundary conditions have been suggested such as D≤πp or D<4p [54]. Therefore, we denoted *p*, 2*p*, π*p* and 4*p* in Figure 5A.

The Odijk regime can be divided into the classic and the backfolded Odijk regimes, which has been intensively studied in previous simulations [50,51,55,56,57,58]. Figure 5A shows that five data points correspond to the backfolded Odijk regime, $2.44\leq D_{eff}/p\leq 3.34$. Microscopic images and intensity profiles can explain the existence of three possible structures such as back-folding [59], S-loop [51,59,60,61] or knot [62,63]. It is relatively challenging to use sequence-specific TAMRA-polypyrrole to investigate DNA conformations from the fluorescent intensity but fortunately, T4 DNA has relatively uniform AT content mostly 60% or more, which fully stained DNA backbone. Furthermore, intensity fluctuation due to AT content is smaller than integrated intensity from the folded and knotted DNA conformations. Furthermore, we often observed the unfolding process from folded conformation right after DNA loading [8,64]. Therefore, after DNA loading, we waited at least five minutes before taking images.

Figure 5B shows intensity profiles to depict double-intensity regions that represent back-folding and triple-intensity regions that represent knot or *S*-loop. For the backfolded Odijk regime, *X*/*L* should be smaller than the prediction from Equation (12) because the existence of back-folding should dramatically reduce DNA stretch. The five data in this regime show that *X*/*L* = 45.4% ± 1.7%, which agreed with our previous simulation for nanochannel confined DNA with an unrelaxed backfolded loop [58]. However, other than folded parts, DNA molecule has unfolded deflection-dominant conformation like the classic Odijk regime. Therefore, if we take short segmental lengths from double and triple intensity regions into account, the length sum from apparent length plus these folded segments would match the deflection dominant length predicted from the gray line (Equation (12)) as illustrated in Figure 5B.

The backfolded Odijk regime requires the formation of hairpins. Previously, Odijk derived an equation for a hairpin radius from the free energy of a hairpin bend for a square nanochannel, which is given by [54]

(13) $$r=\frac{1}{6}\left[{\left(E_{m}^{2}p^{2}+6\sqrt{2}E_{m}Dp\right)}^{1/2}-E_{m}p\right]$$

where *E*_m_ = 1.5071 and *D* was *D*_eff_. Recently, Chen corrected *E*_m_ value as 1.43557 [57]. However, we found that this concept is not appropriate to directly apply to our experiments. For example, Equation (13) predicts r=16 nm for 0.075 mM. Thus, it is impossible to form a hairpin because its diameter would be 2r=32 nm, which is much smaller than p=262 nm and w=224 nm. As illustrated in Figure 5C, a hairpin cannot be formed in 2r≤ w, because the semi-circle representing the electrostatically repulsive region would be located within the radius of *w* from the star-mark, which would not be favorable due to electrostatic repulsion defined by the effective diameter. For 2r=2w, one-third of the semicircle would be in the repulsion region, which may not be favorable to form a hairpin, either. Figure 5A shows a change in the slope at 2r=3w, which is a threshold that separates the classic and the backfolded Odijk regimes. Therefore, we depicted the electrostatically repulsive semi-circle region for 2r=3w in Figure 5C. Compared with two previous cases, it may be possible to form a hairpin because the semi-circle is relatively small.

#### 3.4. Weak-Confinement Regime

The de Gennes theory is well-known to predict the formation of a series of blobs for a polymer in a channel [32], with the polymer stretch given by

(14) $$X/L≅{\left(pw/D^{2}\right)}^{1/3}$$

This scaling relationship predicts nanochannel-confined DNA to shrink and fold by reducing *p* and *w*, which results in the decrease of DNA stretching. However, the theory assumed that the tube diameter should be significantly larger than the monomer size to form a series of blobs within a capillary tube, with each blob behaving like a random coil in a good solvent. Thus, it is questionable whether the low ionic strength conditions in this experiment are suitable for comparison. Nevertheless, there have been some experimental studies performed to evaluate this theory with the similar range of DNA stretches, though they mostly varied nanochannel dimensions, instead of the ionic strength. Those studies reported a little larger scaling exponent (*δ*) for $X\sim D^{-\delta }$ such as 0.86 [65], 0.85 [7], 0.83 [66] or 0.77 [67].

**Figure 6:**
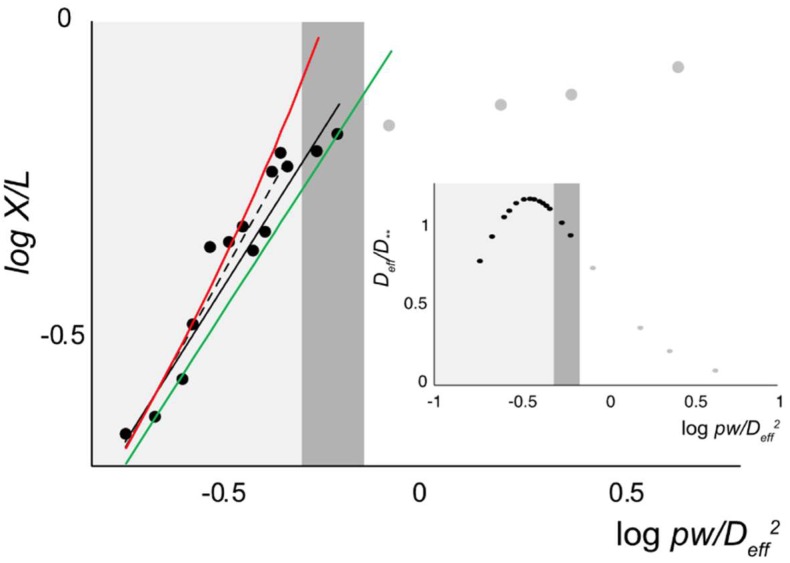
The log-log plot of X/L as a function of $pw/D_{eff}{}^{2}$**.** Gray region includes the de Gennes regime and a dark gray region as the transition regime. The solid line shows the slope = 0.33 (or *δ* = 0.66) with $r^{2}=0.92$ that includes gray and dark gray regions. The dashed line shows the slope = 0.37 (or *δ* = 0.74) with $r^{2}=0.93$. The red line represents Chen’s equation (Equation (16)) [50]. The green line represents the prediction by Werner et al. (Equation (17)) [56]. The inset shows $D_{eff}/D_{**}$ as a function of $pw/D_{eff}{}^{2}$. Gray colored data represent the strong confinement regime.

Figure 6 shows the measured stretch ratio compared to Equation (14) to determine the scaling exponent (*δ*). We used both *D* and *D*_eff_ to see how it affects the scaling exponent. Interestingly, when *D* was used, *δ* was 0.79 (*r^2^* = 0.93), which is consistent with previous studies in which *p* and *w* were fixed [7,65,66]. Interestingly, when *D*_eff_ was used, *δ* was 0.66 as shown in Figure 6, although the linearity was not very high (*r^2^* = 0.92). However, *δ* became 0.74 when we considered only the light gray region with *D*_eff_ (Figure 6). In addition, we found the prefactor to be 1.18 ($D_{eff}>p$), which agreed well with theoretically predicted values of 1.176 [65,68] and 1.046 [69]. We thus obtained the following empirical equation for our measurement in the weak confinement de Gennes regime, which roughly explains the trend of DNA extension with varying the ionic strength.

(15) $$X/L=1.18{\left(pw/D_{eff}{}^{2}\right)}^{0.33}$$

Recently, Chen reported a sophisticated equation particular for small *X*/*L* as given by [50]

(16) $$X/L={\left(pw/D^{2}\chi_{0}C_{0}\right)}^{1/3}$$

where *χ*_0_ and *C*_0_ have the following relationship as

$$\begin{array}{c} \chi_{0}=2e^{2E_{m}p/D}/\sqrt{B_{2}\left(D/p\right)\;}\;\\ C_{0}=\left(1.21\pm 0.01\right){\left(D/p\right)}^{\frac{1}{3}} \end{array}$$

where $E_{m}=1.43557$ and $B_{2}=5.9560$. Alternatively, Werner et al. derived an equation, which is given by [56]

(17) $$X/L=\frac{1}{\sqrt{3}}{\left(\frac{9\sqrt{3}\pi wp}{8D^{2}}\right)}^{1/3}$$

We included these two equations to compare the de Gennes regime as shown as the red and green lines in Figure 6. Here we assumed *D* as *D_eff_* in both Equations (16) and (17).

Finally, we considered the threshold to divide the classical and the extended de Gennes regimes. Previously, Odijk introduced anisotropic blobs within weak confinement regimes, which were referred to as the extended de Gennes regime [31,69]. He defined a threshold as $D_{**}\equiv p^{2}/w$, which represents a blob diameter. Therefore, a blob is anisotropic when $D_{eff}<D_{**}$, which is called the extended de Gennes regime and isotropic when $D_{eff}>D_{**}$, which corresponds to the classical de Gennes regime. Previous experiments and simulation studies investigated these two regimes and the accompanying thresholds [65,69]. However, previous studies used fixed values for *p* and *w*; thus, $D_{**}$ was a constant defining two regimes ($D_{1}<D_{**}<D_{2}$), where $D_{1}$ represents the channel dimension small enough to squeeze blobs for the extended de Gennes regime and $D_{2}$ represents larger channel dimensions capable of providing enough space to form isotropic blobs for the classical de Gennes regime. However, $D_{**}$ changed as a function of ionic strength in our experiment. As shown in Figure 6 inset, the ratio of channel dimension to blob diameter increased to the maximum ($D_{eff}/D_{**}\sim 1$), corresponding to the transition from the classical to the extended de Gennes regimes. This analysis suggests an interpretation that the blob diameters are too large to form within a narrow nanochannel (white region, $D_{eff}<p$ , $D_{eff}/D_{**}<1)$ but the blob size in the other region ($D_{eff}>p$) are approximately the same as the effective channel dimensions ($D_{eff}\;≅\;D_{**}$ ), such that the chain stretch follows de Gennes theory (Equation (15)).

## 4. Conclusions

In this paper, we characterized ionic strength dependent nanochannel confined DNA stretching using TAMRA-polypyrrole instead of YOYO-1. Since YOYO-1 dyes change the persistence length and the contour length, it was difficult to precisely interpret experimental measurement. Contrary to YOYO-1, TAMRA-polypyrrole stained DNA provided both accurate contour length and persistence length. Therefore, using this dye, we first determined ionic strength dependent persistence lengths. For this purpose, we measured flow-induced stretches of surface-tethered DNA by varying ionic strengths. Because the decrease of ionic strength increases both the persistence length and the effective diameter of DNA, DNA stretches increased with the reduction of ionic strength. Our results supported Dobrynin theory that *p* should be inversely proportional to $\sqrt{I}$, instead of *I*. More importantly, our data covered a wide ionic strength range from 0.06 mM to 5.3 mM with a high correlation coefficient ($r^{2}=0.995)$ compared with previous reports. 

Using the ionic strength dependent *p* values, we re-investigated DNA stretches in nanochannels by varying ionic strength from 0.06 mM to 47 mM. We demonstrated that DNA could not enter nanochannel when D≦w at 0.06 mM. Further, our experimental observation confirmed the classic Odijk regime for the full stretch, the backfolded Odijk regime and the de Gennes regime to depict coiled conformations. However, we found some limitations in interpreting our experimental observation with the theories popularly used to explain nanochannel-confined DNA conformations dependent on ionic strengths. For example, swollen DNA with large *w* at low ionic strength did not reach the full extension even when the channel dimension approaches the effective diameter (D→w). There is also a limitation in interpreting DNA folding and knotting using contemporary theories for a hairpin in nanoconfinement. In conclusion, TAMRA-polypyrrole stained DNA provides correct persistence lengths and contour lengths, which can be a powerful means to characterize the statics and dynamics of DNA polyelectrolyte chains confined in nano-/microfluidic devices.

## Figures and Tables

**Table 1:** DNA effective diameter (*w*) calculated from the ionic strength.

IS (mM)	w (nm)	IS (mM)	w (nm)
0.061	250	1.82	38
0.075	224	2.23	34
0.10	193	2.71	31
0.13	164	3.57	27
0.33	98	5.26	22
0.60	70	7.54	18
0.80	60	9.72	16
1.23	47	19.4	11
1.40	44	47.0	7.8
1.59	41	-	-

## Supplementary Materials

The following are available online at http://www.mdpi.com/2073-4360/11/1/15/s1, Figure S1: 16bit clip images. SI figure has raw 16 bit clip images for Figure 4 and Figure 5. ImageJ is recommended for 16 bit image.
