# Supplementary figures and images for: Nanochannel-Confined TAMRA-Polypyrrole Stained DNA Stretching by Varying the Ionic Strength from Micromolar to Millimolar Concentrations
Source: Polymers (Basel). 2018 Dec 22;11(1):15. doi: 10.3390/polym11010015 (PMC6401831; doi:10.3390/polym11010015)

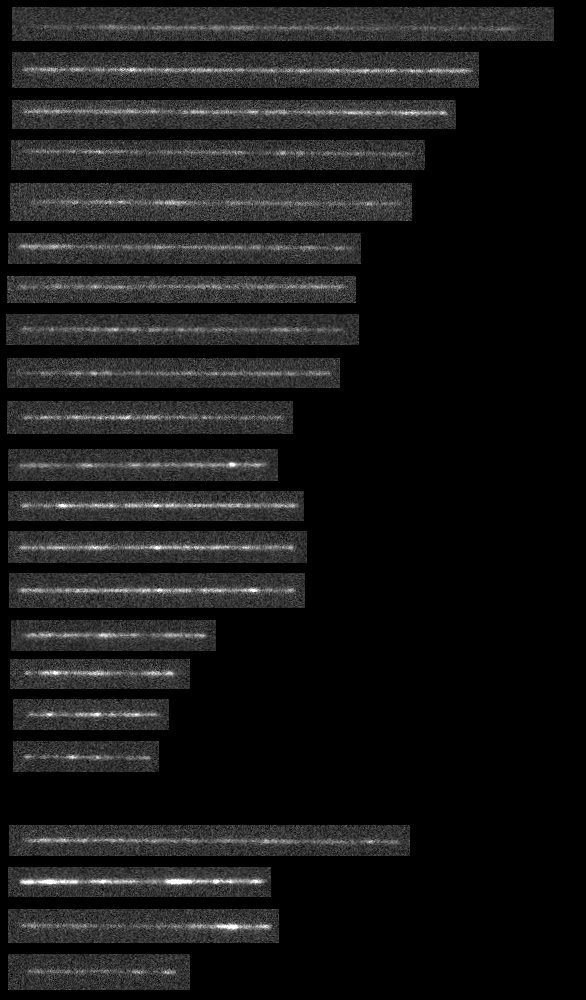

Supplement: Supplementary file 1 [file polymers-11-00015-s001.zip › polymers-377412-supplementary.jpg]
